# Endothelial Insulin Receptors Promote VEGF-A Signaling via ERK1/2 and Sprouting Angiogenesis

**DOI:** 10.1210/endocr/bqab104

**Published:** 2021-05-25

**Authors:** Andrew M N Walker, Nele Warmke, Ben Mercer, Nicole T Watt, Romana Mughal, Jessica Smith, Stacey Galloway, Natalie J Haywood, Taha Soomro, Kathryn J Griffin, Stephen B Wheatcroft, Nadira Y Yuldasheva, David J Beech, Peter Carmeliet, Mark T Kearney, Richard M Cubbon

**Affiliations:** 1 Leeds Institute of Cardiovascular and Metabolic Medicine, The University of Leeds, Leeds LS2 9JT, UK; 2 Imperial College Ophthalmology Research Group, Western Eye Hospital, London NW1 5QH, UK; 3 Laboratory of Angiogenesis and Vascular Metabolism, Center for Cancer Biology, Vlaams Instituut voor Biotechnologie (VIB), Department of Oncology, University of Leuven, Leuven 3000, Belgium

**Keywords:** angiogenesis, endothelial, ERK, insulin, vascular, VEGF

## Abstract

Endothelial insulin receptors (Insr) promote sprouting angiogenesis, although the underpinning cellular and molecular mechanisms are unknown. Comparing mice with whole-body insulin receptor haploinsufficiency (Insr^+/-^) against littermate controls, we found impaired limb perfusion and muscle capillary density after inducing hind-limb ischemia; this was in spite of increased expression of the proangiogenic growth factor *Vegfa*. Insr^+/-^ neonatal retinas exhibited reduced tip cell number and branching complexity during developmental angiogenesis, which was also found in separate studies of mice with endothelium-restricted Insr haploinsufficiency. Functional responses to vascular endothelial growth factor A (VEGF-A), including in vitro angiogenesis, were also impaired in aortic rings and pulmonary endothelial cells from Insr^+/-^ mice. Human umbilical vein endothelial cells with shRNA-mediated knockdown of *Insr* also demonstrated impaired functional angiogenic responses to VEGF-A. VEGF-A signaling to Akt and endothelial nitric oxide synthase was intact, but downstream signaling to extracellular signal-reduced kinase 1/2 (ERK1/2) was impaired, as was VEGF receptor-2 (VEGFR-2) internalization, which is required specifically for signaling to ERK1/2. Hence, endothelial insulin receptors facilitate the functional response to VEGF-A during angiogenic sprouting and are required for appropriate signal transduction from VEGFR-2 to ERK1/2.

Insulin is a primary regulator of systemic carbohydrate and lipid metabolism (1), but also has an important role in vascular function, for example promoting vasodilation and tissue perfusion (2). Indeed, loss of endothelial insulin receptors, or perturbation of their signaling function, induces endothelial dysfunction, hypertension, and atherosclerosis (3-5). Sprouting angiogenesis, the phenomenon of new capillary formation, is another fundamental element of vascular biology that is intrinsically linked to metabolism (6). In this highly orchestrated and conserved process, endothelial “tip cells” emerge from existing vessels, followed by proliferating stalk cells that extend the sprout and form a lumenized vessel; these neo-vessels then anastomose into an immature network that remodels to meet local demands for oxygen and metabolite transport (7). Insulin has been reported to promote angiogenesis in vitro and in vivo; (8-12) these studies found pro-angiogenic effects in nanomolar concentrations in vitro, but did not explore more physiological picomolar concentrations. In vivo, insulin receptor expression is known to be enriched in human tumor endothelial tip cells (13), and loss of endothelial insulin receptors has been shown to impair angiogenesis in murine retinopathy (14). However, it remains unclear how endothelial insulin receptors influence the cellular and molecular processes of angiogenesis and so we set out to define this.

## Materials and Methods

### Animal models

All experimental mice were kept in a conventional animal facility with a 12-hour light/dark cycle and received a standard chow diet. Genotyping was performed using PCR of ear notch (or tail-tip for pups) genomic DNA by Transnetyx (Cordova, TN). All procedures were approved by the Animal Welfare and Ethical Review Committee at the University of Leeds and were conducted in accordance with The Animals (Scientific Procedures) Act of 1986 Amendment Regulations 2012 (SI 2012/3039) under United Kingdom Home Office project licenses PL40/3523 and P144DD0D6.

#### Insulin receptor (Insr) haploinsufficient (Insr^+/-^) mice

As we have previously described (15), Insr^+/-^ mice (also known as IRKO) were obtained from the Medical Research Council Mammalian Genetics Unit (Harwell, Oxfordshire, UK), and were maintained as heterozygotes on a C57BL/6J background. Insr^+/-^ were compared with age-matched wild-type (WT) littermates.

#### Endothelial cell-specific Insr haploinsufficient (ECInsr^+/-^) mice

ECInsr^+/-^ mice were generated by crossing mice that have loxP sites flanking exon 4 of the insulin receptor (Line 006955, The Jackson Laboratory, Bar Harbor, ME) (16) with mice possessing a Cre transgene driven by the Tie2 promoter/enhancer (Line 004128, The Jackson Laboratory) (17) and were maintained on a C57BL/6J background. ECInsr^+/-^ were compared with age-matched Cre-negative Insr^lox/+^ littermates, which are referred to as WT.

### Assessment of retinal developmental angiogenesis

#### Tissue collection and processing

Retinal angiogenesis was assessed in postnatal day 5 pups by precisely following the protocol of Pitulescu et al (18). In brief, all pups from at least 3 litters were included in each experiment, with analysis blinded to the results of genotyping data. Both eyes were processed identically with a mean value from these to represent that pup. Vascular endothelium was stained with Isolectin B4 conjugated with Alexa Fluor 488 (I21411; Thermo Fisher Scientific, Warrington, UK). Costaining with a rabbit anti-mouse anti-Collagen IV antibody (19) followed by an Alexa Fluor-647 conjugated goat anti-rabbit antibody (20) was used to visualize the vascular basement membrane. To define cell proliferation, pups were injected with 125 μg 5-ethynyl-2′-deoxyuridine (EdU) 2 hours before tissue collection; this was stained with Alexa Fluor 647 azide using Click-iT technology (C10640; Thermo Fisher Scientific).

#### Confocal microscopy and image analysis

Microscopy was performed using a Zeiss LSM880 upright confocal microscope with 10×/0.3NA, 20×/0.8NA, and 40×/1.4NA objectives and Zen software (Carl Zeiss Microscopy Ltd, Cambridge, UK). Tile scanning was used to image entire retinal segments with the 20× objective and maximum intensity projection of 5 consecutive 1 Airy unit thickness z-slices was used with the 40× to define tip cells and filopodia. Image analysis used ImageJ (NIH, Bethesda, MD). Radial outgrowth was defined as the distance from the optic disc periphery to the emerging vascular front measured at 12 points in each retina. Vascular area was defined by binary thresholding of the Isolectin B4 signal and expressed as a percentage of the region of interest, bounded either by the peripheral or central half of the vascularized area. Vascular branching was quantified in multiple 200 × 200-μm regions of interest placed between arteries and veins, in the peripheral or central vascular plexus. Tip cell abundance was normalized to the perimeter of the contiguous vascular front in each image and filopodia were normalized to tip cell number. Capillary regression was defined as Collagen IV staining without colocalized Isolectin B4 staining, and expressed as total length per mm^2^ in complete retinal segments, as per the method of Franco et al (21). Endothelial proliferation, defined by EdU^+^ nuclei costaining with Isolectin B4, was quantified in multiple 200 × 200-μm regions of interest placed between arteries and veins, in the peripheral vascular plexus.

### Assessment of pathological angiogenesis after hind-limb ischemia

#### Surgical procedure

Following the protocol we have published (22), 9- to 13-week-old male Insr^+/-^ mice were anesthetized with isoflurane before dissecting the left femoral artery, ligating it proximally at the inguinal ligament and distally at the bifurcation to saphenous and popliteal vessels, and excising the intervening segment.

#### Laser Doppler perfusion imaging

Laser Doppler analysis (Moor LDI2-HR, Moor Systems, UK) of ischemic and nonischemic limbs was performed postoperatively in a temperature-controlled environment, to confirm induction of ischemia, and repeated weekly until day 21. Images were analyzed (MoorLDI software, Version 5.3, Moor Systems, UK) to derive an ischemic to nonischemic limb perfusion ratio, based upon flux below the level of the inguinal ligament.

#### Tissue collection and processing

Ischemic and contralateral gastrocnemius muscle was harvested and fixed in 4% paraformaldehyde for 48 hours, whereas adductor muscles were snap frozen with liquid nitrogen for RNA isolation. Fixed muscle specimens were embedded in optimal cutting temperature media (Tissue-Tek OCT compound, Sakura, Netherlands) before snap freezing in liquid nitrogen and cryosectioning at 10-µm thickness. Vascular endothelium was stained with Isolectin B4 conjugated with Alexa Fluor 488 (I21411; Thermo Fisher Scientific) and slides were mounted with DAPI-Fluoromount-G (Southern Biotech, AL) to define nuclei.

#### Confocal microscopy and image analysis

Microscopy was performed using a Zeiss LSM880 upright confocal microscope with 20×/0.8NA objective and Zen software (Carl Zeiss Microscopy Ltd). Image analysis used ImageJ (NIH). Vascular area was defined by binary thresholding of the Isolectin B4 signal and expressed as a percentage of the image area.

### RT-PCR

Snap frozen ischemic adductor muscle was mechanically lysed in Trizol reagent (Sigma Aldrich, Gillingham, UK) to isolate RNA. After reverse transcription to generate cDNA (kit), quantitative PCR was performed (ABI Prism 7900HT, Applied Biosystems) using Taqman probes against murine vegfa (Mm01281449-m1), murine insr (Mm00439688_m1), murine actb (Mm00607939_s1), 18s (Mm01281449-m1); 18s or actb were used to normalize gene expression using the equation


$$\begin{array}{l} 2^{-deltaCT}\times \;100. \end{array}$$

### Ex vivo aortic ring angiogenesis

Aortae were harvested from 8- to 12-week-old Insr^+/-^ mice under terminal isoflurane anesthesia and then processed according to the protocol of Baker et al (23). In brief, after dissection of perivascular fat and overnight storage in serum free OptiMEM media (Thermo Fisher Scientific), aortae were cut in to 1-mm-thick rings that were then embedded in rat type I collagen. Rings were incubated for 5 days at 37°C in 5% CO_2_ in Opti-MEM media containing 2.5% fetal calf serum (FCS), 50 ng/mL VEGF-A_165_ (R&D Systems, Abingdon, UK) and penicillin-streptomycin, with a media change on day 3. Rings were then fixed with 4% paraformaldehyde, stained with BS-1 lectin-fluorescein (Sigma Aldrich) to define endothelium, and then imaged with an inverted confocal microscope (LSM700, Carl Zeiss Microscopy Ltd.); tiled images were collected using a 10×/0.2NA objective and stitched using Zen software. Image analysis was performed with Image J (NIH), defining the number of fluorescein staining sprouts per ring and the mean length of these sprouts; mean data were then produced for each experimental animal from at least 4 rings.

### Mouse pulmonary endothelial cell studies

#### Isolation and functional analysis

Pulmonary endothelial cells (PECs) were isolated from both lungs of 8- to 12-week-old Insr^+/-^ mice, precisely following the protocol of Sobczak et al (24). This uses immuno-magnetic selection of CD31^+^ cells, which are then cultured in EGM2 media (Lonza, Slough, UK) for 10 to 14 days before a second round of immuno-magnetic selection from ICAM2^+^ endothelial cells that were cultured for a further 5 to 7 days in EGM2 before functional assays.

#### Matrigel sprouting assay

Twenty-four well plates were coated with growth factor reduced Matrigel (BD Biosciences, Wokingham, UK) before seeding each well with 2 × 10^5^ PEC suspended in EBM2 media (Lonza) containing 1% FCS and 50 ng/mL of VEGF-A_165_. After 24 hours, phase contrast microscopy was used to image each well and count tubule-like structures according to our published protocol (22). Each sample was run in triplicate, with a single mean datapoint calculated for each experimental animal.

#### Scratch wound assay

PECs were grown to confluence in EGM2 media on 1% gelatin coated 96-well plates before forming a scratch wound using the WoundMaker tool (Essen Bioscience, Royston, UK) and imaging wound closure hourly in a live cell imaging system (Incucyte, Essen Bioscience) to define residual wound area.

#### Boyden chamber

Following our published protocol (25), 5 × 10^4^ PECs were seeded in 1% gelatin coated Boyden chamber apparatus to define migration toward 50 ng/mL VEGF-A_165_. The number of migrating cells per microscopic field was counted using standard light microscopy and presented as net migration by subtracting the number of cells migrating in paired control experiments without VEGF-A_165_ gradient.

#### Cell proliferation

Sparsely seeded PECs on 1% gelatin coated plastic, cultured in EGM2 media, were exposed to 10 µM EdU 2 hours before fixation with 4% paraformaldehyde and processing with the Click-iT EdU cell proliferation assay (Thermo Fisher Scientific) to label nuclei containing actively forming DNA with Alexa Fluor 488 and a Hoechst nuclear counterstain. Confocal microscopy (LSM700, Carl Zeiss Microscopy Ltd.) was used to define the proportion of EdU^+^ nuclei.

### Human umbilical vein endothelial cell studies

#### Cell culture and lentiviral manipulation

Human umbilical vein endothelial cells (HUVECs; PromoCell, Heidelberg, Germany) were cultured at 37°C in 5% CO_2_ in EGM2 media on 1% gelatin-coated plasticware and used between passages 3 and 6. Silencing of the insulin receptor was induced using insulin receptor shRNA introduced by lentiviral particles (SHCLNV-NM_00208, TRCN0000196786; MISSION, Sigma Aldrich), with GFP-targeting shRNA lentivirus particles (SHC002H; MISSION, Sigma Aldrich) serving as control. Both lentiviruses were applied at 15 multiplicity of infection and HUVECs were used in downstream experiments 4 days after transduction.

#### Bead sprouting assay

Following the protocol of Nakatsu et al (26), HUVECs were coated on to the surface of Cytodex-3 microcarrier beads (Sigma Aldrich) and then embedded in a fibrin matrix that was overlaid with EGM2 media (Lonza) without the supplemental bullet kit, but containing 50 ng/mL human VEGF-A_165_ (PeproTech, NJ) and 5ng/ml human basic FGF (PeproTech). After 48 hours of incubation at 37°C in 5% CO_2_, 25 beads per condition were imaged with phase contrast microscopy (Olympus CX41, Olympus Life Sciences, Southend-On-Sea, UK) and analyzed with Image J (NIH), defining sprouts per bead and the mean length of these sprouts; mean data were then produced for each experimental condition.

#### Scratch wound assay

HUVECs were grown to confluence in EGM2 media on 1% gelatin-coated 96-well plates before forming a scratch wound using the WoundMaker tool (Essen Bioscience) and imaging wound closure 8 hours later to define percentage wound closure from baseline.

#### Adhesion assay

HUVECs were seeded on to 1% gelatin-coated 24-well plates in EBM2 media with 1% FCS, with or without 50 ng/mL VEGA-A_165_, at a density of 4 × 10^4^ cells per well and left for 1 hour before washing 3 times with PBS and fixing with 4% paraformaldehyde. Cells were counterstained with Hoechst and Phalloidin Alexa Fluor 488 conjugate and imaged with confocal microscopy (LSM700, Carl Zeiss Microscopy Ltd.) to count adherent cells per mm^2^.

### Assessment of VEGF signaling

#### Western blotting

HUVECs were lysed in cell extraction buffer (FNN0011; Thermo Fisher Scientific; containing, in mmol/L, 10 mM Tris, pH 7.4, 100 mM NaCl, 1 mM EDTA, 1 mM EGTA, 1 mM NaF, 20 mM Na4P2O7, 2 mM Na3VO4, 1% Triton X-100, 10% glycerol, 0.1% SDS, 0.5% deoxycholate, 2 sodium orthovanadate, 0.5 μg/mL leupeptin, 0.2 PMSF, 0.5 μg/mL aprotinin). Cell extracts were centrifuged for 15 minutes before protein measurement using the biocinochinic acid assay (Thermo Fisher Scientific). Equal amounts of protein were resolved on SDS-polyacrylamide gels (Thermo Fisher Scientific) and transferred to polyvinyldine difluoride membranes. Immunoblotting was carried out with primary antibodies against beta-actin (27), insulin receptor-beta (28), Akt (29), phospho-S473 Akt (30), endothelial nitric oxide synthase (eNOS) (31), phospho-S1177 eNOS (32), extracellular signal-regulated kinase 1/2 (ERK1/2) (33), phospho-T202/Y204 ERK1/2 (34), vascular endothelial growth factor receptor 2 (VEGFR2) (35), and phospho-Y951 VEGFR2 (36). Blots were incubated with appropriate peroxidase-conjugated secondary antibodies and developed with enhanced chemiluminescence (37, 38), and imaged with SynGene chemiluminescence imaging system (SynGene, Cambridge, UK). Densitometry of phospho-proteins was normalized to respective total proteins from the same sample and then these data were normalized to the value of unstimulated control shRNA-transduced cells in each experiment (which included paired control and insulin receptor shRNA-transduced cells, with samples run on 1 membrane). VEGF-A induced signaling was calculated by subtracting the data from unstimulated (control or insulin receptor shRNA transduced) cells from their respective VEGF-A–exposed cells in each experiment.

#### Surface biotinylation and immunoprecipitation of VEGFR2

Surface VEGFR2 biotinylation, immunoprecipitation, and VEGFR2 Western blotting were performed according to our previously published protocol (39). Briefly, HUVECs were incubated for 1 hour at 4°C with 0.5 mg/mL biotin sulfo-NHS (Sigma-Aldrich, Gillingham, UK), before being stimulated with 50 ng/mL VEGF at 37°C for either 5 or 15 minutes. At the end of the exposure period, the cells were washed 3 times with PBS with calcium and magnesium before either: immediate lysis with cell extraction buffer to enable the measurement of total VEGFR2 in the sample; or, treated with 0.5 mL of 0.05% trypsin/EDTA to cleave and remove any remaining biotin-labelled, cell surface VEGFR2, meaning detected biotin-labelled VEFGR2 would define only internalized protein. The trypsinized cell pellet was lysed using cell extraction buffer as before. Immunoprecipitation of VEGFR2 was carried out using protein A Dynabeads (Thermo Fisher Scientific) loaded with anti-VEGFR2 antibody (diluted 1:100) (35) for 30 minutes with rotation at room temperature. The beads were washed with radio-immunoprecipitation assay buffer to remove any unbound antibody before incubating with cell lysate for 1 hour with rotation at room temperature to allow the biotinylated antigen-antibody complexes to form. At the end of the pull-down period, the beads were washed 5 times with radio-immunoprecipitation assay buffer (50 mmol/L Tris-HCl [pH 8.0], 150 mmol/L NaCl, 0.5% [w/v] sodium deoxycholate, 0.1% [w/v] SDS, and 1% [v/v] Igepal) to remove any nonspecific binding. The immunoprecipitated, biotinylated VEGFR2 complexes were mixed with dissociation buffer and boiled to release the complexes from the beads. The proteins were resolved by electrophoresis through 4% to 12% polyacrylamide gels and then transferred to nitrocellulose membrane. The membrane was blocked for 1 hour in PBS (1.5 mmol/L KH2PO4, 2.7 mmol/L Na2HPO4, 150 mmol/L NaCl [pH 7.4]) containing 5% (w/v) dried milk powder and 0.1% (v/v) Tween-20, followed by incubation with peroxidase-conjugated streptavidin (1:1000 dilution in PBS containing 0.1% [v/v] Tween-20) for 1 hour. Bound peroxidase conjugates were visualized using an enhanced chemiluminescence detection system (Amersham Biosciences). Quantification of immunoblots was performed using ImageJ software.

### Statistics

All data are presented as mean (SEM). Comparison between groups was performed using Student *t* tests, or 2-way ANOVA for time series data. All tests were 2-sided and statistical significance was defined as *P* < 0.05.

## Results

### Pathological angiogenesis is impaired in Insr^+/-^ mice and is associated with impaired responsiveness to VEGF

**Figure 1.**
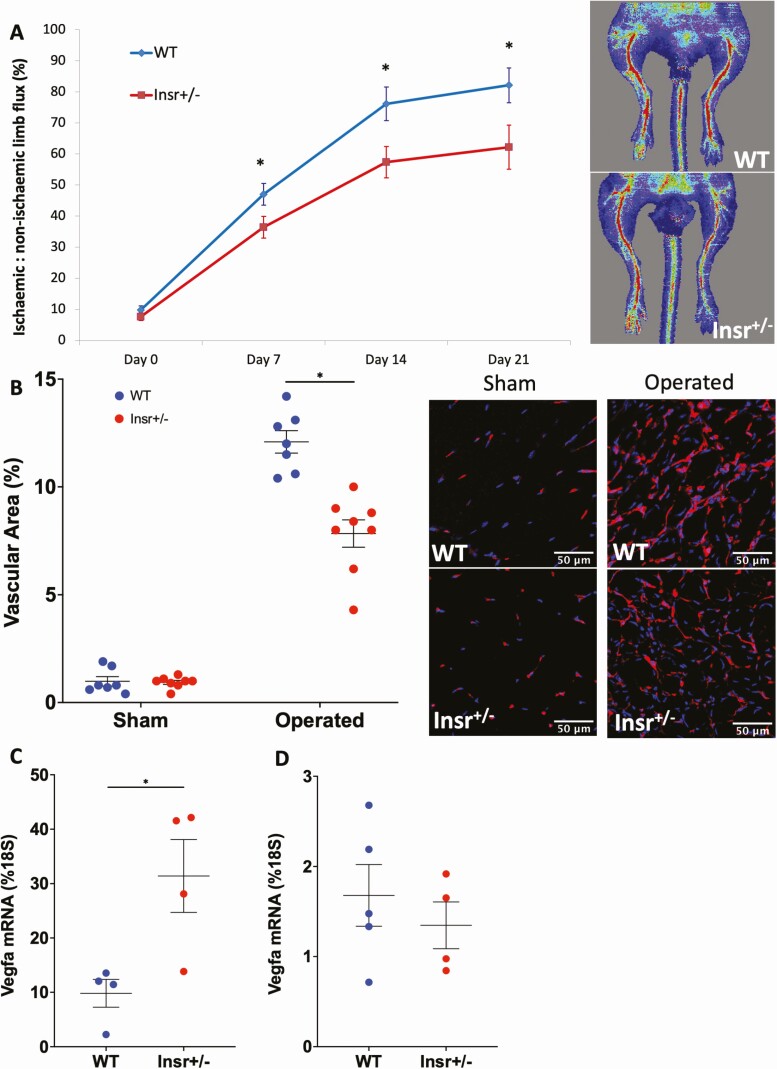
Angiogenesis is impaired in Insr^+/-^ mice with hindlimb ischemia. (A) Ischemic to nonischemic limb perfusion defined by laser Doppler imaging, with representative day 21 images, showing impaired recovery from hindlimb ischemia in Insr^+/-^ vs WT (n = 14, 13). (B) Confocal immunofluorescence of ischemic and nonischemic gastrocnemius muscle reveals reduced capillary density in ischemic Insr^+/-^ vs WT muscle. Representative images of ischemic muscle show isolectin B4 stained capillaries in red and nuclei in blue. Scale bars denote 50 μm. (n = 8, 7). (C) *Vegfa* mRNA normalized to *18S* mRNA is higher in the ischemic limb adductor muscle of Insr^+/-^ vs WT (n = 4, 4). **D**) *Vegfa* mRNA normalized to *18S* mRNA is higher in the nonischemic limb adductor muscle of Insr^+/-^ vs WT (n = 4, 4). *P < 0.05. Insr, insulin receptor; WT, wild-type.

**Figure 2.**
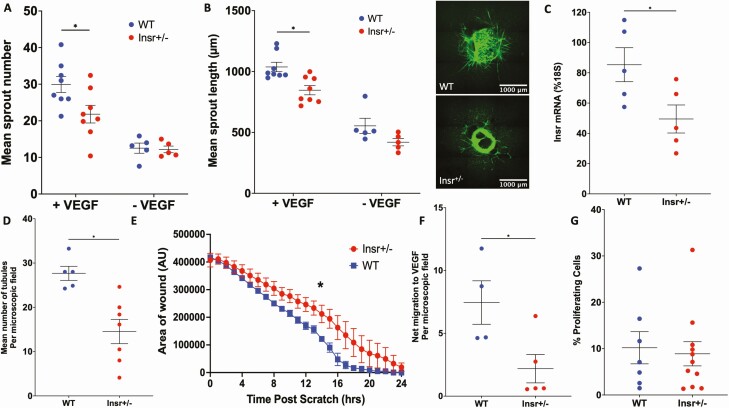
Insr^+/-^ exhibits impaired in vitro functional responses to VEGF. Capillary sprouting from aortic rings embedded in a collagen matrix with VEGF-A_165_ is reduced in Insr^+/-^ vs WT (A), as is mean sprout length (B); representative images show isolectin B4 staining of endothelium in green, with scale bars denoting 1000 μm (n = 5, 5). (C) Insulin receptor (*Insr*) mRNA normalized to *18S* mRNA is reduced in Insr^+/-^ vs WT PEC (n = 5, 5). (D) In vitro angiogenesis in Matrigel is impaired in Insr^+/-^ vs WT PEC (n = 7, 4). (E) Scratch wound closure is impaired in Insr^+/-^ vs WT PEC (n = 5, 6). (F) Migration toward VEGF-A_165_ in Boyden chamber apparatus is impaired in Insr^+/-^ vs WT PEC (n = 5, 4). (G) Proliferation defined by nuclear EdU incorporation is similar in Insr^+/-^ and WT PEC (n = 11, 7). *P < 0.05. EdU, 5-ethynyl-2′-deoxyuridine; Insr, insulin receptor; PEC, pulmonary endothelial cell; VEGF, vascular endothelial growth factor; WT, wild-type.

To study pathological angiogenesis, we induced hindlimb ischemia in Insr^+/-^ mice and quantified limb perfusion recovery every 7 days using laser Doppler imaging. This revealed that, in spite of similar reductions in limb perfusion immediately postoperatively, Insr^+/-^ exhibited lower ischemic limb perfusion at all timepoints thereafter (Fig. 1A), being 82% (5.6) in WT and 62% (7.1) in Insr^+/-^ (*P* < 0.05) at day 21. Histological analysis of gastrocnemius muscle also revealed a lower capillary density in the ischemic limb of Insr^+/-^ at day 21 (vascular area 12% [0.5] vs 8% [0.6] in WT and Insr^+/-^, respectively, *P* < 0.05 Fig. 1B), in spite of greater *VEGFA* mRNA in the ischemic limb adductor muscle of Insr^+/-^ (31.4% [6.7] of *18S* mRNA in Insr^+/-^ vs 9.8% [2.5] WT *P* < 0.05; Fig. 1C). Notably, *Vegfa* mRNA was similar in the nonischemic limb adductor muscle or WT and Insr^+/-^ (1.7% [0.3] of *18S* mRNA in WT vs. 1.4% [0.3] in Insr^+/-^; Fig. 1D). The increased ischemic muscle expression of *Vegfa* is indicative of significant residual ischemia and could also imply an inadequate functional response to this central regulator of angiogenesis. To address this possibility, we explanted aortae from a separate group of Insr^+/-^ mice to embed rings in a collagen matrix containing VEGF-A_165_, which induces sprouting angiogenesis. This revealed fewer capillary sprouts emerging from Insr^+/-^ aortae in the presence of VEGF (30 [2.2] in WT vs 22 [2.4] in Insr^+/-^; *P* < 0.05; Fig. 2A), and the length of sprouts was also reduced (1037 µm [39] WT vs 847 µm [38] Insr^+/-^; *P* < 0.05; Fig. 2B). Next, we isolated PECs for functional studies; these exhibited appropriate reduction in *Insr* mRNA (85% [5] of *18S* in WT vs 49% [4] in Insr^+/-^; *P* < 0.05; Fig. 2C). We then conducted a Matrigel in vitro angiogenesis assay, which demonstrated reduced tubule formation in Insr^+/-^ (28 [2] tubules per microscopic field in WT vs 14.5 [2.4] in Insr^+/-^; *P* < 0.05; Fig. 2D). Similarly, a scratch wound assay performed on confluent PECs revealed significantly slower closure of the wound formed in Insr^+/-^ PECs (area under curve 4,430,670 arbitrary units WT [154,516] vs 5,085,825 [126,748] arbitrary units Insr^+/-^; *P* < 0.05; Fig. 2E). Because the Matrigel and scratch wound assays define responses to VEGF-A with other stimulatory factors, we then performed assays to more specifically define functional responses to VEGF-A. First, we conducted a migration assay using Boyden chamber apparatus and found fewer Insr^+/-^ PECs migrated toward VEGF-A_165_ (7.5 [1.7] WT vs 2.2 [1.1] Insr^+/-^ net cell migration to VEGF per microscopic field; *P* < 0.05; Fig. 2F). Second, we studied VEGF-A_165_–induced PEC proliferation using EdU incorporation and elicited no difference between Insr^+/-^ and WT (10.2% [3.5] WT vs 8.9% [2.6] Insr^+/-^ EdU^+^ cells; *P* = 0.77; Fig. 2G). Collectively, these data imply that Insr^+/-^ endothelial cells have selectively impaired migratory responses to VEGF-A.

### Developmental angiogenesis is impaired in Insr^+/-^ and ECInsr^+/-^ mice

**Figure 3.**
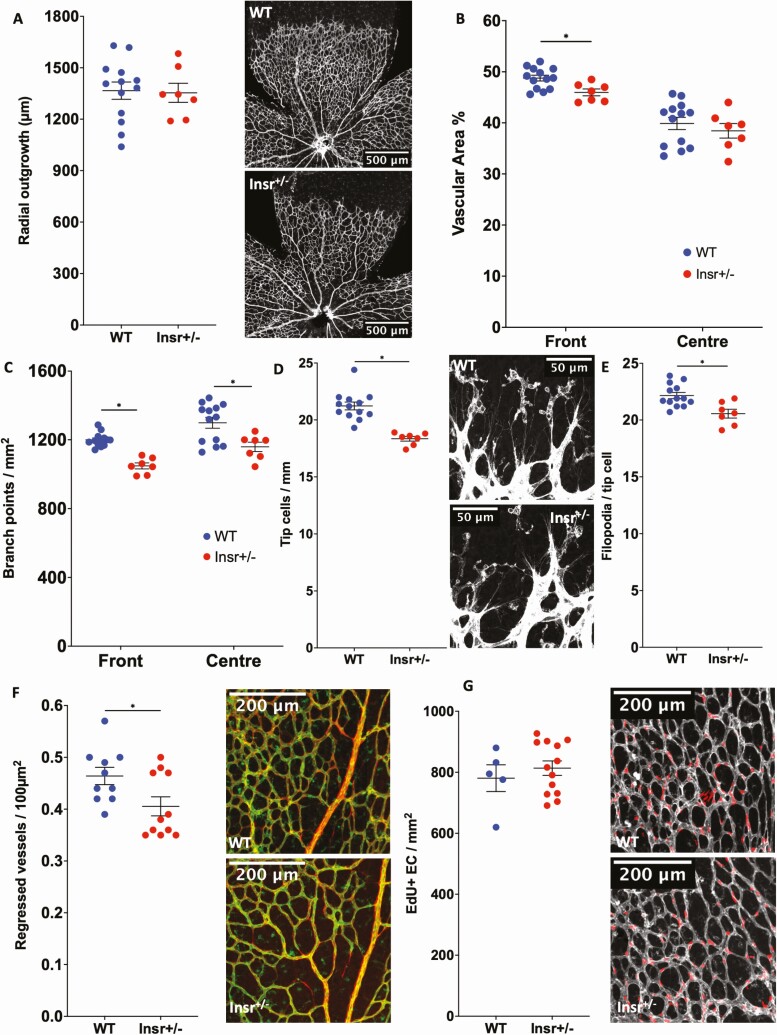
Developmental angiogenesis is impaired in the neonatal P5 retina of Insr^+/-^ mice. (A) Radial outgrowth of the developing retinal vasculature is comparable in Insr^+/-^ and WT, with representative images showing white isolectin B4 staining of endothelium and scale bars denoting 500 μm (n = 7, 13). (B) Vascular endothelial area is reduced in the peripheral half of the retinal vasculature in Insr^+/-^ vs WT (n = 7, 13). (C) Vascular branching is reduced in the peripheral and central zones of the retinal vasculature in Insr^+/-^ vs WT (n = 7, 13). (D) Emerging tip cells per millimeter of vascular front perimeter are reduced in Insr^+/-^ vs WT, with representative images showing white isolectin B4 staining of endothelium and scale bars denoting 50 μm (n = 7, 13). (E) The number of filopodia per tip cell is similar in Insr^+/-^ vs WT (n = 7, 13). (F) The number of regressed vessels, defined as Collagen IV sleeves (red) without overlying isolectin B4 (green) in representative images, is lower in Insr^+/-^ than WT (n = 11, 10). (G) The number of proliferating endothelial cells, defined as EdU^+^ nuclei (red) overlying isolectin B4 (white) in representative images, is similar in Insr^+/-^ and WT (n = 13, 5). *P < 0.05. EdU, 5-ethynyl-2′-deoxyuridine; Insr, insulin receptor; WT, wild-type.

Next, we asked whether the abnormalities of pathological angiogenesis in Insr^+/-^ were recapitulated during developmental angiogenesis, which we assessed using whole-mounted retinas, at P5 when the vasculature is still developing. The radial outgrowth of the retinal vascular plexus was similar in Insr^+/-^ and WT (1367 µm [50] WT vs 1354 µm [56] Insr^+/-^, *P* = 0.87; Fig. 3A), although there was reduced vascular area in the peripheral vascular plexus of Insr^+/-^ (48.8% [0.6] WT vs 46.0% [0.7] Insr^+/-^, *P* < 0.05; Fig. 3B) and reduced vascular branching complexity (front 48.0 [0.4] WT vs 41.9 [0.7] Insr^+/-^ branches per microscopic field, *P* < 0.05; center 52.0 [1.3] WT vs 46.4 [1.1] Insr^+/-^ branches per microscopic field, *P* < 0.05; Fig. 3C) in both central and peripheral vascular plexuses of Insr^+/-^. High-resolution images of the emerging vascular plexus revealed a reduction in the number of sprouting tip cells of Insr^+/-^ (21.2 [0.3] WT vs 18.3 [0.2] Insr^+/-^ tip cells per mm, *P* < 0.05; Fig. 3D), fewer filopodia per length of vascular forefront (22.0 [0.5] WT vs 18.1 [0.3] Insr^+/-^ filopodia per 100 µm, *P* < 0.05), and fewer filipodia per tip cell (22.1 [0.3] WT vs 20.6 [0.4] Insr^+/-^ filopodia per tip cell, *P* < 0.05; Fig. 3E). Because reduced vascularity could also be explained by increased vessel regression, we quantified the number of “empty” collagen IV sleeves (ie, collagen IV basement membrane without overlying Isolectin B4, linking 2 regions of established vasculature) in the retinal periphery; we observed fewer in Insr^+/-^ vs WT, indicating regression was not exaggerated (0.46 [0.02] WT vs 0.40 [0.02] Insr^+/-^ regressed vessels/100 µm^2^; *P* = 0.028; Fig. 3F). Next, we asked if endothelial cell (EC) proliferation was reduced in Insr^+/-^, but found similar numbers of EdU^+^ ECs in the peripheral retinal vasculature of both genotypes (781 [44] WT vs 814 [24] Insr^+/-^ EdU^+^ EC per mm^2^; *P* = 0.53; Fig. 3G). Overall, these data are compatible with a reduction in vascular sprouting in the emerging vasculature of Insr^+/-^, resulting in a less branched neovasculature.

**Figure 4.**
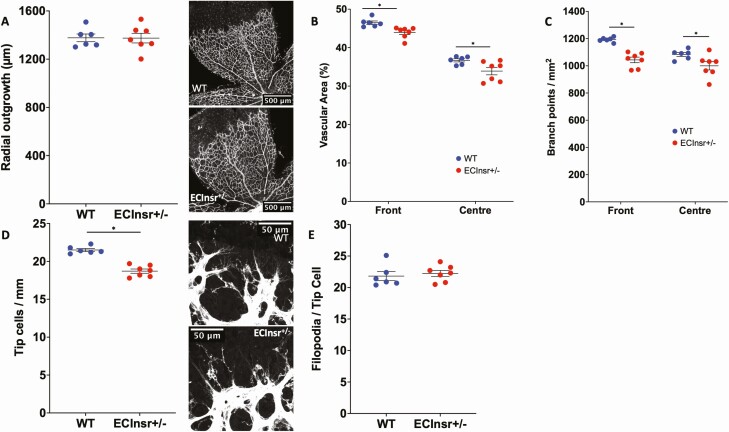
Developmental angiogenesis is impaired in the neonatal P5 retina of ECInsr^+/-^ mice. (A) Radial outgrowth of the developing retinal vasculature is comparable in ECInsr^+/-^ and WT, with representative images showing white isolectin B4 staining of endothelium and scale bars denoting 500 μm (n = 7, 6). (B) Vascular endothelial area is reduced in the peripheral and central zones of the retinal vasculature in ECInsr^+/-^ vs WT (n = 7, 6). (C) Vascular branching is reduced in the peripheral and central zones of the retinal vasculature in ECInsr^+/-^ vs WT (n = 7, 6). (D) Emerging tip cells per millimeter of vascular front perimeter are reduced in ECInsr^+/-^ vs WT, with representative images showing white isolectin B4 staining of endothelium and scale bars denoting 50 μm (n = 7, 6). (E) The number of filopodia per tip cell is similar in ECInsr^+/-^ vs WT (n = 7, 6). ECInsr, endothelial cell insulin receptor; WT, wild-type.

To discern whether loss of insulin receptors expressed by ECs contribute to the retinal vascular phenotype of Insr^+/-^, we then studied mice with ECInsr^+/-^. Again, the radial outgrowth of their retinal vascular plexus was similar to controls (1377 µm [32] WT vs 1375 µm [40] ECInsr^+/-^; *P* = 0.96; Fig. 4A), although there was reduced vascular area (front 46.5% [0.5] WT vs 44.0 [0.5] ECInsr^+/-^, *P* < 0.05; center 36.7% [0.4] WT vs 33.9% [1.0] ECInsr^+/-^, *P* < 0.05; Fig. 4B) and branching complexity (front 1192.5 [7.0] WT vs 1043.3 [20.4] ECInsr^+/-^ branch points per mm^2^, *P* < 0.05; center 1083.5 [14.3] WT vs 1000.0 [30.5] ECInsr^+/-^, *P* < 0.05; Fig. 4C) in both the peripheral and central vascular plexuses of ECInsr^+/-^. Also mirroring Insr^+/-^ phenotype, there was a reduction in the number of sprouting tip cells of ECInsr^+/-^ (21.5 [0.2] WT vs 18.7 [0.3] ECInsr^+/-^ tip cells/mm; *P* < 0.05; Fig. 4D), along with fewer filopodia per length of vascular forefront (21.2 [0.4] WT vs 18.7 [0.3] ECInsr^+/-^ filopodia per 100 µm; *P* < 0.05), but with no difference in filopodia per tip cell between genotypes (21.8 [0.7] WT vs 22.2 [0.5] ECInsr^+/-^ filopodia per tip cell; *P* = 0.65; Fig. 4E). Collectively, these data suggest that endothelial cell insulin receptor expression is important in the generation of vascular sprouts, and the branching structure of the nascent vasculature.

### Insulin receptor silencing impairs human EC functional responsiveness to VEGF

**Figure 5.**
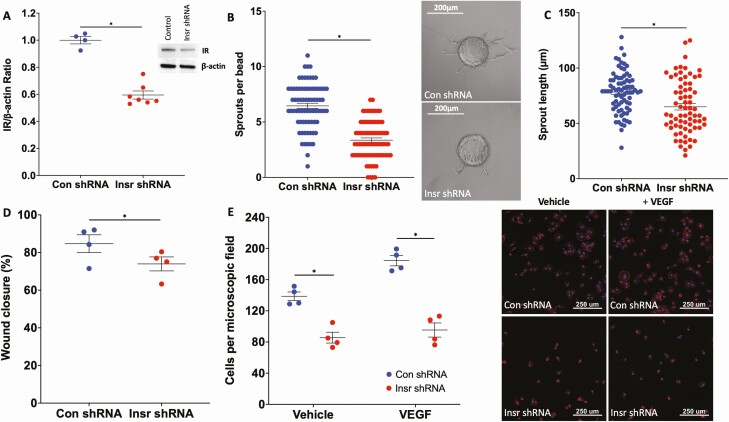
Insr knockdown in HUVECs impairs functional responses to VEGF. (A) Insulin receptor protein knockdown of 40% was achieved in Insr shRNA HUVECs vs control shRNA HUVECs, with representative gel (n = 7, 7). (B) Angiogenic sprout numbers were reduced from Cytodex beads coated with Insr shRNA HUVECs vs control shRNA HUVECs, with representative microscopy images (n = 3, 3). (C) Angiogenic sprout length was similar from Cytodex beads coated with Insr shRNA HUVECs vs control shRNA HUVECs (n = 3, 3). (D) Scratch wound closure was impaired in Insr shRNA HUVECs vs control shRNA HUVECs (n = 4, 4). (E) Adhesion to gelatin was impaired in Insr shRNA HUVECs vs control shRNA HUVECs, especially in context of media supplemented with VEGF-A_165_; representative microscopy images show DAPI-defined nuclei in blue and phalloidin-defined filamentous actin in red, with scale bars denoting 250 μm (n = 4, 4). *P < 0.05. HUVEC, human umbilical vein endothelial cell; Insr, insulin receptor; VEGF, vascular endothelial growth factor.

To explore the relevance of these data in human ECs, we transduced HUVECs with lentivirus particles to deliver insulin receptor targeting shRNA (referred to as Insr shRNA), or control GFP-targeting shRNA (referred to as control [Con] shRNA), reducing insulin receptor protein by 40% (Fig. 5A). Transduced HUVECs were then coated onto Cytodex-3 carrier beads and embedded in a fibrin matrix to study VEGF-A_165_–induced sprouting angiogenesis in vitro. Mirroring data from Insr^+/-^ aortic ring sprouting experiments (Fig. 2A-C), we observed that Insr shRNA HUVECs produced fewer sprouts (6.5 [1.2] Con shRNA vs 3.4 [1.0] Insr shRNA sprouts per bead; *P* < 0.05; Fig. 5B), although the mean length of sprouts was unaffected (78.3 µm [10.7] Con shRNA vs 65.0 µm [14.0] Insr shRNA, *P* > 0.05; Fig. 5C). Insr shRNA HUVECs exhibited impaired scratch wound closure (84.7% [4.7] Con shRNA vs 74.0% [3.7] Insr shRNA; *P* < 0.05; Fig. 5D) and impaired adhesion to gelatin coated plates, which was more marked in the presence of VEGF-A_165_ (vehicle 138.7 [5.5] Con shRNA vs 85.6 [6.9] Insr shRNA cells per microscopic field; *P* < 0.05; VEGF-A_165_ 184.3 [6.6] Con shRNA vs 95.4 [9.1] Insr shRNA cells per microscopic field; *P* < 0.05; Fig. 5E). Overall, these data suggest that the insulin receptor is also important for VEGF-A–induced angiogenic sprouting and cell motility in human EC.

### Endothelial insulin receptors are required for VEGFR2 internalization and subsequent ERK signaling

**Figure 6.**
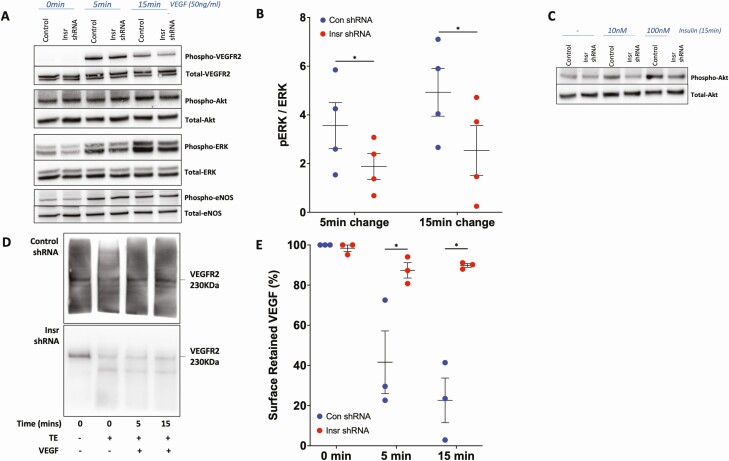
Insr knockdown in HUVECs impairs signaling responses to VEGF. (A) Representative blots illustrating major VEGF signaling nodes in Insr shRNA HUVECs and control shRNA HUVECs grown on gelatin at baseline, 5 minutes, and 15 minutes after stimulation with VEGF-A_165_ 50 ng/mL, (B) with quantification of VEGF-induced phosphorylation of ERK1/2 at 5 and 15 minutes (n = 4, 4). (C) Representative blot illustrating impaired insulin-stimulated Akt phosphorylation in Insr shRNA HUVECs vs control shRNA HUVECs after 15 minutes’ exposure to 10 and 100 nm insulin. (D) Representative blots and (E) quantification from surface biotinylation experiment illustrating impaired internalization of VEGFR2 in Insr shRNA HUVECs vs control shRNA HUVECs (n = 4, 4). (D) Detected biotin-labelled VEGFR2 in the presence or absence of TE and so is not directly represented in €, which presents derived internalization data. HUVEC, human umbilical vein endothelial cell; Insr, insulin receptor; TE, trypsin exposure; VEGF, vascular endothelial growth factor; VEGFR2, vascular endothelial growth factor receptor 2.

Because we had demonstrated that insulin receptor expression influenced functional responses to VEGF-A in human and murine ECs, we then asked if this was associated with altered VEGF-A signaling. VEGF-A promotes angiogenesis by binding to VEGFR2, a cell membrane-bound receptor tyrosine kinase that initiates a complex intracellular signaling cascade. We therefore stimulated Insr shRNA and control shRNA HUVECs with 50 ng/mL VEGF-A_165_ and studied major VEGF-A signaling nodes 5 and 15 minutes later, along with unstimulated cells (Fig. 6A). Insr shRNA HUVECs exhibited unaffected activation of VEGFR2 (measured by phosphorylation at Y951), or the downstream nodes Akt (measured by phosphorylation at S473) and eNOS (measured by phosphorylation at S1177) (data not shown). However, downstream activation of ERK1/2 (measured by phosphorylation at T202/Y204) was impaired in Insr shRNA HUVECs at 5 and 15 minutes after stimulation (5-minute change: 3.6 [0.95] Con shRNA vs 1.9 [0.53] Insr shRNA pERK/ERK ratio; *P* < 0.05; 15-minute change: 4.9 [0.98] Con shRNA vs 2.5 [1.02] Insr shRNA pERK/ERK ratio; *P* < 0.05; Fig. 6B). Control experiments defining the signaling response to insulin revealed clear reduction in Akt activation after 15 minutes, confirming that Insr shRNA induced the expected changes in activation of this major insulin signaling node (Fig. 6C). Importantly, the activation of Akt and ERK1/2 downstream of VEGFR2 follows highly distinct pathways, with internalization of VEGFR2 being essential for only the latter (40); moreover, integrins are known to influence this process (41). Hence, we then asked whether VEGF-A induced internalization of VEGFR2 was impaired in Insr shRNA HUVEC by performing a surface biotinylation assay to quantify surface-resident and internalized VEGFR2 protein. This revealed that, although baseline surface located VEGFR2 was similar in both groups, there was reduced VEGFR2 internalization in Insr shRNA HUVECs (5 minutes: 41.6% [15.6] Con shRNA vs 87.4% [3.8] Insr shRNA surface-retained VEFGR2; *P* < 0.05; 15 minutes: 20.6% [12.9] Con shRNA vs 89.8% [0.9] Insr shRNA surface-retained VEFGR2; *P* < 0.05; Fig. 6D,E), which is known to selectively impede ERK1/2 activation. Overall, these data reveal a selective deficit in VEGF-A signal transduction in Insr shRNA HUVECs, which is likely to result from impaired internalization of activated VEGFR2.

## Discussion

### Major findings and implications

**Figure 7.**
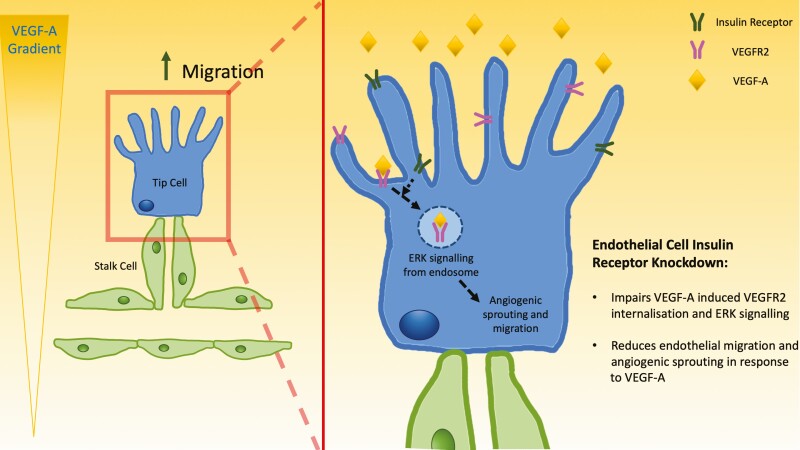
Schematic illustration of the proposed role of Insr during sprouting angiogenesis. Insr expression is known to be enriched in tip ECs, which migrate along VEGF gradients, leading emerging sprouts during angiogenesis. Knockdown of Insr in ECs impairs VEGF signaling to ERK1/2 as a result of impaired VEGFR2 internalization, which manifests as diminished sprout formation and EC migration. EC, endothelial cell; ERK, extracellular signal-regulated kinase; Insr, insulin receptor; VEGF, vascular endothelial growth factor; VEGFR2, vascular endothelial growth factor receptor 2.

Our data reveal for the first time that endothelial insulin receptors are required for appropriate migration and angiogenic sprouting in response to VEGF-A, both in vitro and in vivo. At a molecular level, we found that insulin receptor expression promotes the internalization of VEGF-A–activated VEGFR2, allowing signaling to ERK1/2. Our data suggest that the proangiogenic effects of insulin receptors relate to crosstalk with VEGF-A signaling, although the nature of this interaction, and whether insulin participates in the process, requires further study (Fig. 7). This previously unappreciated crosstalk establishes a further link between systems regulating metabolism and angiogenesis.

### Insulin and angiogenesis

A number of studies have shown insulin exerts proangiogenic effects, although they did not dissect the role of endothelial insulin receptors (8-12). These mainly in vitro studies revealed proangiogenic effects of nanomolar range insulin, but did not explore more physiological picomolar concentrations. The extent to which picomolar insulin augments ERK signaling in endothelial cells is a source of disagreement in the literature (11, 42-44), probably reflecting known heterogeneity between endothelial populations, including in their insulin receptor expression. Notably, insulin receptor expression is reported to be enriched in endothelial tip cells of human tumors (13), yet tip cells generally lack a lumen (45), so may be exposed to lower concentrations of insulin than other endothelial cells. Hence, one possible explanation for our data is a ligand-independent role of tip cell insulin receptors. Indeed, recent data from Ronald Kahn’s group indicate a ligand-independent role of insulin receptors in the membrane trafficking of brown pre-adipocytes (46), potentially aligning with our findings. However, although the concentrations of insulin experienced by sprouting endothelial cells in vivo are unknown, our in vitro data are likely to reflect low picomolar concentrations of insulin because of its presence in fetal calf serum. This may suggest a role for insulin in promoting VEGF-induced ERK signaling, as has been shown for epidermal growth factor signaling (47). Another explanation may be that insulin regulates a common endocytic mechanism for its own receptor and VEGFR2, as discussed later.

The only existing data describing the role of endothelial insulin receptors in angiogenesis were published by Kahn’s group in 2003. Using mice with complete deletion of endothelial insulin receptors, they found a 57% reduction in retinal neovascularization during oxygen-induced retinopathy (14). However, they did not study the individual cellular processes contributing to angiogenesis, or examine VEGF signaling. Our in vivo data suggest that insulin receptors regulate endothelial tip cell emergence and migration, although with no major impact on endothelial cell proliferation. Notably, we found no reduction in nonischemic muscle tissue vascularity of Insr^+/-^ mice, implying that impaired vascularization ultimately catches up; this is seen in many published examples of impaired angiogenesis (48-50), presumably reflecting persistent activation of proangiogenic programmes.

### VEGFR2 signaling

VEGF-A binding to VEGFR2 induces a complex intracellular signaling cascade (51), a crucial element of which is internalization (endocytosis) of ligand-bound VEGFR2. This moves the phosphorylated receptor to a domain where it is less susceptible to the phosphatase PTP1B, hence sustaining signal transmission, which is particularly important for ERK1/2 signaling (40). VEGFR2 internalization, and subsequent ERK1/2 signaling, is known to be crucial to vascular biology (eg, during arterial morphogenesis and lymphatic specification) (52, 53). Although less well studied, ERK1/2 activation has recently emerged as regulating endothelial tip cell function and sprouting angiogenesis; (54, 55) importantly, tip cells are exposed to the highest VEGF-A concentrations during angiogenesis, and tip cell ERK phosphorylation is prevented in vivo by VEGF inhibition (7, 55). There are many known interacting partners of VEGFR2, which can modify its signaling and internalization (41, 56). The insulin receptor signaling adaptor, insulin receptor substrate-1, has been implicated in receptor endocytosis (57), and is reported to interact with VEGFR2 (58). Interestingly, recent data show that insulin signaling to ERK1/2 (and Src homology phosphatase 2) feeds back via serine phosphorylation of insulin receptor substrate-1 to induce insulin receptor internalization, which augments ERK1/2 signaling (59). Therefore, it is possible that VEGFR2 internalization is similarly affected, and this putative insulin-dependent mechanism also warrants further exploration. Integrins are also well established to modulate the propagation of ERK1/2 signals downstream of many growth factor receptors, including during angiogenesis, so warrant future assessment (60). Finally, the insulin receptor can regulate cytoskeletal actin remodeling (61), another process that influences endocytosis (62), warranting further exploration in future.

### Limitations

Although we show that internalization of activated VEGFR2 is impaired, the underlying mechanism of this requires further investigation. It will also be interesting to explore signal transduction downstream of other receptor tyrosine kinases to assess the generalizability of this phenomenon. As alluded to earlier, it is also important to acknowledge that impaired VEGFR2 internalization may not be the only mechanism by which Insr silencing impairs VEGF-A responses; indeed, intracellular signaling networks are highly complex, as is their perturbation. Moreover, our work only sought to describe the fundamental links between insulin receptors and VEGF signaling during angiogenesis, and so we cannot comment on disease relevance. However, obesity-induced insulin resistance is associated with reduced vascular insulin receptor expression and impaired angiogenesis (63), so it would be interesting to explore endothelial VEGFR2 internalization and ERK signal transduction in this setting. Finally, our ECInsr^+/-^ control data come from littermates expressing Tie2-Cre, and recent data show that Cre is not biologically inert (64); however, the similar retinal vascular phenotype of Insr^+/-^ and ECInsr^+/-^ mice provides some reassurance that off-target Cre effects do not underpin our findings.

## Conclusions

We show that endothelial insulin receptors are required for appropriate migration and angiogenic sprouting in response to VEGF-A, along with internalization of activated VEGFR2 and downstream signaling to ERK1/2. This novel link between major regulators of systemic metabolism and angiogenesis warrants further mechanistic exploration to understand its wider relevance.

## Data Availability

All datasets generated during and/or analyzed during the current study are not publicly available but are available from the corresponding author on reasonable request.

## Abbreviations

Con: control
EC: endothelial cell
EdU: 5-ethynyl-2′-deoxyuridine
eNOS: endothelial nitric oxide synthase
ERK: extracellular signal-regulated kinase
FCS: fetal calf serum
HUVEC: human umbilical vein endothelial cell
Insr: insulin receptor
PEC: pulmonary endothelial cell
VEGF: vascular endothelial growth factor
VEGFR2: vascular endothelial growth factor receptor 2
WT: wild-type

## Additional Information

***Disclosures:*** The authors have nothing to disclose.
